# Medical conditions, social and neurological patients of the T-lymphotropic virus humanities-HTLV I

**DOI:** 10.1186/1742-4690-11-S1-P35

**Published:** 2014-01-07

**Authors:** GLC Gaspar, MAS Souza, T Masteguim, S Matta, J Casseb, GS Olival, R Marcusso, ACP Oliveira

**Affiliations:** 1 Institute of Infectious Diseases Emilio Ribas, IIER, São Paulo, SP, Brazil

## Introduction

The virus Human T lymphotropic (HTLV) can affect neurological disorders, gastrointestinal motility at low, causing constipation and nutritional deficiencies may occur. To identify the nutritional status of patients with HTLV I and social factors, clinical and neurological that may interfere with their quality of life.

## Methods

Retrospective analysis, with individuals in a group of 150 patients who have regular monitoring in the outpatient HTLV, this group were evaluated 42 adult patients of both sexes, attended in 2012. We evaluated anthropometric factors (gender, age and body mass index - BMI), social (practical exercise), clinical and neurological (bowel movement and ambulation, neurological and vitamin D) and presence of coinfection. Nutritional status was determined by BMI (WHO, 2000). Data were analyzed using SPSS.

## Results

**Figure 1:**
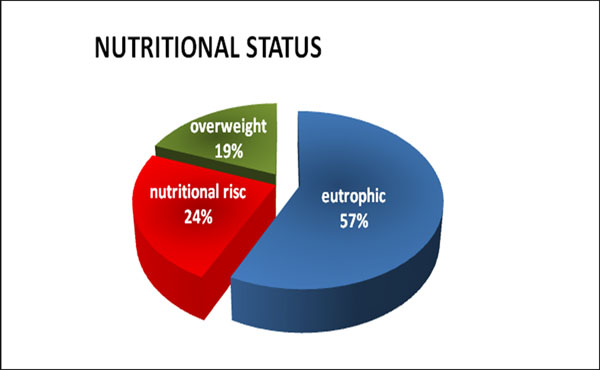
Distribution of patients with HTLV I, according to nutritional status (BMI). IIER, 2013 (n = 42).

**Figure 2:**
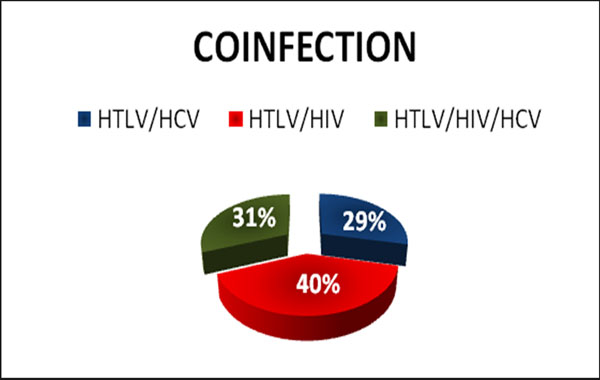
Distribution of patients with HTLV I, according to the presence of co-infections (HIV, HCV and HTLV). IIER, 2013 (n = 42).

Patients with a mean age of 49 years, 55% male, 62% eutrophic (Figure 1). Observed 40% of HIV coinfected patients (Figure 2). Identified 65% of the sample with constipation, 59.5% showed no neurological signs, 61.9% had difficulty walking; 74.6% were sedentary and 52.4% had vitamin D insufficiency (Table 1). There was a statistically significant correlation with nutritional diagnosis and HIV coinfection of HTLV p = 0.005.

**Table 1:** Social characteristics, clinical and neurological patients with HTLV I, Emilio Ribas Institute of Infectious Diseases, 2013 (n=42).

Variables	n	%
Bowel Habit		35%
Habit - Constipation	28	**65%**

Practical exercise - YES	11	25,4%
Practical exercise - NOT	31	**74,6%**

Vitamin D deficiency (below 10ng/ml)		47,6%
Vitamin D insufficiency (10-30ng/ml)	22	**52,4%**

Neurological disorders - YES	17	40,5%
Neurological disorders - NOT	25	**59,5%**

Difficulties in Ambulation - YES	26	**61,9%**
Difficulties in Ambulation	16	38,1%

## Conclusion

Population eutrophic observed low motility and ambulation, vitamin D insufficiency, in HIV coinfection and physical inactivity, which are factors that influence the health and quality of life.

